# Supplementary material for: Diabetes self-management education interventions in the WHO African Region: A scoping review
Source: PLoS One. 2021 Aug 17;16(8):e0256123. doi: 10.1371/journal.pone.0256123 (PMC8370626; doi:10.1371/journal.pone.0256123)
Supplement: S2 Appendix — (DOCX) [file pone.0256123.s002.docx]

| **S2 Appendix: Sample search strategy (Embase)** | |
| --- | --- |
| **#** | **Searches** |
| 1 | exp Diabetes mellitus/ |
| 2 | (Type 1 diabet* OR type I diabet* OR insulin dependent diabet*).mp. [mp=title, abstract, original title, name of substance word, subject heading word] |
| 3 | (Type 2 diabet* OR type II diabet* OR non-insulin dependent diabet*).mp. [mp=title, abstract, original title, name of substance word, subject heading word] |
| 4 | # 1 AND #2 AND #3 |
| 5 | limit 4 to (English language and yr = "2000-2019") |
| 6 | (Self-management education OR self-support education OR self-care education) AND (program* OR intervention).mp. [mp=title, abstract, original title, name of substance word, subject heading word] |
| 7 | #5 AND #6 |
| 8 | limit 7 to (English language and yr = "2000-2019") |
| 9 | (Sub-Saharan Africa OR developing countr* OR low and middle-income countr* OR resource poor setting).mp. |
| 10 | limit 9 to (English language and yr = "2000-2019") |
| 11 | # 8 AND #10 |
