# Supplementary material for: Diabetes self-management education interventions in the WHO African Region: A scoping review
Source: PLoS One. 2021 Aug 17;16(8):e0256123. doi: 10.1371/journal.pone.0256123 (PMC8370626; doi:10.1371/journal.pone.0256123)
Supplement: S3 Appendix — (DOCX) [file pone.0256123.s003.docx]

| **S3 Appendix: Quality assessment tool used to evaluate the methodological quality of the included studies** | | | |  |
| --- | --- | --- | --- | --- |
| **Rating Component** | **Description** | **Rating** | | |
|  |  | Strong = 3 | Moderate = 2 | Weak = 1 |
| Selection Bias | 1. Whether study participants are likely to be representative of the target population 2. The percentage of selected individuals who agreed to participate in the study |  |  |  |
| Study Deign | 1. Whether the study design was indicated 2. For randomized controlled trials, whether the method of randomization was described. If yes, whether the method was appropriate |  |  |  |
| Confounders | 1. Whether there were significant differences between groups prior to the intervention 2. If yes, whether there was an indication of the percentage of relevant confounders that were controlled (either in the design or analysis) |  |  |  |
| Blinding | 1. Whether there were significant differences between groups prior to the intervention 2. If yes, whether there was an indication of the percentage of relevant confounders that were controlled (either in the design or analysis) |  |  |  |
| Data Collection Methods | 1. Whether there were significant differences between groups prior to the intervention 2. If yes, whether there was an indication of the percentage of relevant confounders that were controlled (either in the design or analysis) |  |  |  |
| Withdrawals & Drop-Outs | 1. Whether there were significant differences between groups prior to the intervention 2. If yes, whether there was an indication of the percentage of relevant confounders that were controlled (either in the design or analysis) |  |  |  |

See <http://www.city.hamilton.on.ca/phcs/EPHPP/> for more information on the Effective Public Health Practice Project Quality Assessment Tool
