# Supplementary material for: Diabetes self-management education interventions in the WHO African Region: A scoping review
Source: PLoS One. 2021 Aug 17;16(8):e0256123. doi: 10.1371/journal.pone.0256123 (PMC8370626; doi:10.1371/journal.pone.0256123)
Supplement: S4 Appendix — (DOCX) [file pone.0256123.s004.docx]

| **S4 Appendix: Quality assessment of the included studies** | | | |  |  | |  |  |
| --- | --- | --- | --- | --- | --- | --- | --- | --- |
| **Study** | **Methodological quality** | | | | | | | |
|  | **Selection Bias** | **Study Deign** | **Confounders** | **Blinding** | **Data Collection Methods** | **Withdrawals & Drop-Outs** | | **Overall rating** |
| Assah et al. [25] | 3 | 2 | 2 | 1 | 2 | | 3 | 2.17 - Moderate |
| Bett [26] | 2 | 1 | 1 | 1 | 1 | | 2 | 1.33 - Weak |
| Debussche et al. [27] | 3 | 3 | 2 | 2 | 3 | | 3 | 2.67 - Strong |
| Gill et al. [28] | 2 | 1 | 2 | 2 | 3 | | 1 | 1.83 - Moderate |
| Hailu et al. [29] | 2 | 2 | 2 | 2 | 2 | | 1 | 1.83 - Moderate |
| Hailu et al. [30] | 2 | 2 | 2 | 2 | 2 | | 2 | 2.00 - Moderate |
| Mash et al. [31] | 1 | 3 | 1 | 1 | 3 | | 2 | 1.83 - Moderate |
| Muchiri et al. [32] | 2 | 3 | 2 | 2 | 2 | | 2 | 2.17 - Moderate |
| Afemikhe & Chipps [33] | 1 | 2 | 1 | 1 | 2 | | 1 | 1.33 - Weak |
| Essien et al. [34] | 2 | 3 | 2 | 2 | 1 | | 2 | 2.00 - Moderate |
| Park et al. [35] | 2 | 1 | 1 | 1 | 1 | | 1 | 1.17 - Weak |
| Asante et al. [36] | 2 | 2 | 2 | 1 | 1 | | 3 | 1.83 - Moderate |
| Price et al. [37] | 1 | 2 | 2 | 1 | 1 | | 1 | 1.33 - Weak |
| Amendezo et al. [38] | 2 | 1 | 2 | 2 | 2 | | 1 | 1.67 - Moderate |
| Muchiri et al. [39] | 2 | 3 | 2 | 2 | 2 | | 2 | 2.17 - Moderate |
| MakkiAwouda et al. [40] | 2 | 1 | 1 | 1 | 1 | | 1 | 1.17 - Weak |
| Baumann et al. [41] | 2 | 2 | 2 | 1 | 2 | | 1 | 1.67 - Moderate |
| van der Does & Mash [42] | 1 | 1 | 2 | 1 | 1 | | 2 | 1.33 - Weak |
| Gathu et al. [43] | 2 | 2 | 1 | 1 | 2 | | 1 | 1.50 - Weak |
